# Hydrothermal Synthesis of Iridium-Substituted NaTaO_3_ Perovskites

**DOI:** 10.3390/nano11061537

**Published:** 2021-06-10

**Authors:** David L. Burnett, Christopher D. Vincent, Jasmine A. Clayton, Reza J. Kashtiban, Richard I. Walton

**Affiliations:** 1 Department of Chemistry, University of Warwick, Coventry CV4 7AL, UK; d.burnett@bham.ac.uk (D.L.B.); cdv1230@gmail.com (C.D.V.); jasmine.clayton@warwick.ac.uk (J.A.C.); 2 Department of Physics, University of Warwick, Coventry CV4 7AL, UK; r.jalilikashtiban@warwick.ac.uk

**Keywords:** perovskite, tantalate, crystallisation, nanocrystals, photocatalysis, water splitting

## Abstract

Iridium-containing NaTaO_3_ is produced using a one-step hydrothermal crystallisation from Ta_2_O_5_ and IrCl_3_ in an aqueous solution of 10 M NaOH in 40 vol% H_2_O_2_ heated at 240 °C. Although a nominal replacement of 50% of Ta by Ir was attempted, the amount of Ir included in the perovskite oxide was only up to 15 mol%. The materials are formed as crystalline powders comprising cube-shaped crystallites around 100 nm in edge length, as seen by scanning transmission electron microscopy. Energy dispersive X-ray mapping shows an even dispersion of Ir through the crystallites. Profile fitting of powder X-ray diffraction (XRD) shows expanded unit cell volumes (orthorhombic space group *Pbnm*) compared to the parent NaTaO_3_, while XANES spectroscopy at the Ir L_III_-edge reveals that the highest Ir-content materials contain Ir^4+^. The inclusion of Ir^4+^ into the perovskite by replacement of Ta^5+^ implies the presence of charge-balancing defects and upon heat treatment the iridium is extruded from the perovskite at around 600 °C in air, with the presence of metallic iridium seen by in situ powder XRD. The highest Ir-content material was loaded with Pt and examined for photocatalytic evolution of H_2_ from aqueous methanol. Compared to the parent NaTaO_3_, the Ir-substituted material shows a more than ten-fold enhancement of hydrogen yield with a significant proportion ascribed to visible light absorption.

## 1. Introduction

The hydrothermal synthesis of ABO_3_ perovskite oxides has attracted a large amount of interest in the past decade [1]. This includes families of materials with important properties such as titanates (B = Ti) with dielectric properties [2], piezoelectric zirconate-titanates (B = Zr, Ti) [3], multiferroic chromites (B = Cr) [4], and ferrites (B = Fe) with applications in redox catalysis [5]. The synthesis of this range of compositions work builds on a body of literature on hydrothermal crystallisation of one of the prototypical perovskites BaTiO_3_ [6]. The attraction of the hydrothermal synthesis method lies in the use of solution chemistry to enable crystallisation from solution directly at mild temperatures typically less than 200 °C: this allows adjustment of the crystal morphology, including size and shape of crystallites on the nanoscale, as well as the possibility of isolating compositions not stable under more extreme conditions [7,8,9,10,11,12]. This level of control in the synthesis of oxide materials is lacking in traditional high temperature routes, and even in co-precipitation or sol–gel approaches, in which a firing step is needed to induce crystallinity; this means annealing takes place, with control of crystallite size being difficult to achieve.

Many functional oxide materials have been accessed via hydrothermal synthesis routes, some with unique properties arising from their nanostructure. A notable example is the formation of nanowires of Cu_2_O that have considerably enhanced photoactivity in the visible region of the spectrum [13]. The hydrothermal method also provides a convenient method for the formation of composite materials, where the growth of an oxide on a support in situ provides new functional solids with exceptional properties. Examples include the formation of graphene oxide conjugated Cu_2_O nanowires for gas sensing [14], ammonia sensing from Cu_2_O nanoparticles decorated with MoS_2_ nanosheets [15], and graphene oxide–MnO_2_ nanocomposites for supercapacitors [16].

Niobate and tantalate perovskites, ANbO_3_ and ATaO_3_ where A = Na or K, have attracted much attention due to their practical applications in two important areas: as potential lead-free electroceramics, as end-members of the piezoelectric material K_0_._5_Na_0_._5_NbO_3_, and as photocatalysts for applications such as water splitting and carbon dioxide conversion. Hydrothermal synthesis of these materials has been extensively investigated and the pathways during the formation of the perovskite products have been mapped [17,18,19]. Some control of crystallite morphology has proved possible; for example, it has been shown that a low concentration of Nb_2_O_5_ as a precursor leads to nanorods or nanoplates of NaNbO_3_, while a lower concentration of NaOH yields cubes [20]. Crystallite morphology of NaNbO_3_ can also be influenced by the choice of niobium oxide precursor [21], the pH of the solution [22], and the choice of solvent [23]. For electroceramics, fine-grained ceramics can be produced by annealing the powders from hydrothermal reactions: piezoelectric ceramics formed from hydrothermally prepared alkali niobates and tantalates have shown characteristics comparable to ceramics made by conventional methods, but with the advantage of lower sintering temperatures to achieve densification [24,25,26]. For photocatalysis, the high surface areas of the nanostructured crystallites offers high reactivity; for example, Shi et al. prepared nanocubes and compared them with nanowires, and found a correlation between crystal shape and photocatalytic activity for hydrogen evolution from water/methanol [27]. High surface areas are also useful for support materials for co-catalysts.

In photocatalysis, NaNbO_3_ and NaTaO_3_ are typically doped with substituent metals to tune their band gaps, and then used as supports for precious metals with the particular aim to permit absorption of visible light [28]. Doping of perovskite oxides with precious metal cations is of more general interest, but can present a synthetic challenge as there is a strong tendency for the precious metal substituent to be reduced to the elemental state, even upon heating in air. LaCr_1−*x*_M*_x_*O_3_ (LCMO) (*x* = 0.01, 0.05, 0.10 with M = Pd, Co, Ir) materials were prepared using conventional solid-state synthesis from single metal oxide precursors at 1200 °C, with a reduction in band gap observed for all substituted materials [29]. Rh-doped BaTiO_3_ nanoparticles, with 2% of the substituent, were prepared by a co-precipitation route using oxalate as solution additive at temperatures between 700 and 900 °C [30]. The Rh was found in the +3 oxidation state and on heating to higher temperatures the rhodium was lost, with phase transformation of the perovskite. 0.5% iridium-doped SrTiO_3_ was prepared by a solid-state method, and in a second step under reducing conditions, exsolution of the Ir was found that resulted in supported Ir nanoparticles, embedded in the oxide support, showing little agglomeration when used in CO oxidation catalysis [31]. Ir-doped SrTiO_3_ (1–5% Ir) has also been studied for photocatalysis, and the oxidation state of the Ir was found to dictate the properties towards photocatalytic water splitting, with Ir^4+^ giving the most favourable properties [32]. Kudo et al. studied a number of precious-metal-doped perovskites for photocatalytic water splitting; in the case of NaNbO_3_ and NaTaO_3_, prepared by a solid-state method, doping with Ir or Rh creates band gaps suitable for visible light absorption when co-doped with alkaline earth or lanthanide cations, Ba and La, respectively [33,34].

Given the need for convenient synthesis methods for precious-metal-substituted perovskites, which may also allow control of the substituent oxidation state, we have investigated the use of hydrothermal reaction conditions. In this paper we consider the possibility of iridium substitution in NaTaO_3_ by a direct hydrothermal synthesis and show that a one-step crystallisation is able to form nanocubes with homogeneous distribution of iridium, as proven by various experimental techniques. We chose this composition to study since there is already a body of work on the hydrothermal synthesis of NaTaO_3_, and the case of iridium substitution has been reported by other synthesis methods, which provide a comparison to the milder solution conditions. To our knowledge, the inclusion of precious metal substituents in an oxide perovskite host by hydrothermal synthesis has not yet been reported. Our aim was to explore the maximum level of inclusion of the precious metal in the perovskite host structure to test the synthetic strategy. We present an assessment of the use of the materials as visible light photocatalysts for hydrogen evolution from water.

## 2. Materials and Methods

### 2.1. Materials Synthesis

Chemicals used were sourced from chemical companies: Ta_2_O_5_ (Alfar Aesar, Heysham, UK, 99%), hydrated IrCl_3_ (Johnson Matthey, London, UK, 52.29% Ir), NaOH (Fisher Scientific, Loughborough, UK, Laboratory Grade) and H_2_O_2_ (Sigma Aldrich, Gillingham, UK, 30% in water by volume). The substituted tantalates were synthesised by the reaction of 1.1 mmol tantalum (V) oxide in 10 mL of 10 M NaOH in 40 vol% H_2_O_2_, with a portion of the oxide replaced by a chosen amount of iridium (III) chloride. The use of H_2_O_2_ as an oxidant was based on our previous work on hydrothermal synthesis of iridium oxides to prevent the formation of metallic iridium [35]. After stirring the reagents for one hour in a 20 mL polytetrafluoroethylene container, the reaction mixture was sealed in a steel autoclave and heated at 240 °C for three days. After cooling naturally to room temperature, the powders were collected via vacuum filtration, and washed with 20 mL of 3 M HNO_3_, followed by 20 mL of acetone, and then dried in air at 70 °C before further study.

### 2.2. Characterisation

Powder X-ray diffraction (XRD) data were recorded using a Panalytical Empyrean diffractometer (Malvern Panalytical, Malvern, UK) equipped with a Cu target, giving Cu Kα1/2 radiation. Data were recorded in reflection, Bragg–Brentano geometry from samples in silicon plates. The powder diffraction patterns were analysed using the GSAS-II software [36], with Pawley or Rietveld fits performed using published crystal structures of NaTaO_3_ as a starting point to refine lattice parameters. Powder X-ray thermodiffractometry was performed using a Bruker D8 instrument (Bruker AXS Ltd., Coventry, UK) with Cu Kα1/2 radiation and fitted with an Anton Paar XRK 900 chamber (Anton Paar GmbH, Graz, Austria) and a VÅNTEC solid-state detector (Bruker AXS Ltd., Coventry, UK); this allowed heating of a sample from room temperature to 900 °C and XRD patterns were recorded at intervals of 50 °C. Before each data collection, the temperature was allowed to equilibrate for 5 min.

Scanning electron microscopy was performed using a ZEISS SUPRA 55-VP FEGSEM scanning electron microscope (Oberkochen, Germany) using a field emission gun with an accelerating voltage between 5 and 20 kV and fitted with an Oxford Instruments (Abingdon, UK) energy-dispersive X-ray spectroscopy (EDS) spectrometer that allows elemental composition analysis

Scanning transmission electron microscopy (STEM) was performed using a JEOL ARM200Fdouble aberration corrected instrument (Welwyn Garden City, UK) operating at 200 kV. Specimens were dispersed by ultrasound in ethanol and dropped onto 3 mm lacey carbon grids supplied by Agar Scientific (Stansted, UK). Annular dark field STEM (ADF-STEM) images were obtained using a JEOL annular field detector at a probe current of ~23 pA with a convergence semi-angle of ~25 mrad. Energy-dispersive X-ray spectroscopy (EDS) measurements were carried out with an Oxford Instruments X-MaxN100TLE windowless silicon drift detector (Abingdon, UK) to determine the elemental composition and distribution. The program clTEM [37] was utilised to produce simulations of ADF-STEM images based on the crystal model oriented at (010) zone axis over an area of 1.5 nm by 2.6 nm. Inelastic phonon scattering was applied using the frozen phonon approximation method via an iterative approach to resemble molecular deviation from its equilibrium position under the electron probe at room temperature. The following thermal parameters (<*u*^2^>) were used during simulation: 0.0166 A^2^ Na, 0.0048 A^2^ Ta, and 0.0080 A^2^ for O [38].

X-ray absorption near-edge spectroscopy (XANES) spectra at the iridium L_III_-edge were recorded using Beamline B18 of the Diamond Light Source, Harwell, UK [39]. Data were collected in transmission mode from samples diluted with appropriate amounts of polyethylene powder (~20% sample by mass) and pressed into self-supporting discs around 1 mm thick. Incident energies were selected using a water-cooled, fixed-exit, double-crystal monochromator with Si(111) crystals. The beam was focused horizontally and vertically using a double toroidal mirror, coated with Pt, 25 m from the source, while a pair of smaller plane mirrors were used for harmonic rejection. The raw data were normalised using the software ATHENA (version 0.9.26) [40] to produce XANES spectra.

Diffuse reflectance spectroscopy was performed on powder samples using a Shimadzu UV-2600i UV-Vis spectrophotometer (Milton Keynes, UK). A barium sulfate standard was used as a baseline for the measurements.

The photocatalytic hydrogen production was performed in a Pyrex glass vessel with a top quartz window for vertical illumination in a closed-gas circulation system. In a typical run, 45 mg of catalyst was suspended in 20 vol% methanol solution in water. Then, 1.0 wt.% of Pt was loaded on the photocatalyst particles via photodeposition as a cocatalyst, to provide H_2_ evolution sites. The glass reactor vessel was then sealed and repeatedly vacuumed by a rotary pump and purged with argon gas to remove the residual air. Subsequently, the reactor was irradiated with an 800 W Xe-Hg lamp (Newport, RI, USA) from the top, with full-spectrum intensity of 200 mW cm^−2^. Experiments were also performed with visible light (420 nm cut-off filter) with intensity of 100 mW cm^−2^. The infrared component in the radiation was removed by a circulating water filter. The temperature in the reactor was maintained at 25 °C by external water circulation. The amount of generated H_2_ gas was quantitatively analysed every 2 h by a gas chromatograph (Shimadzu GC-2014; Molecular sieve 5A, TCD detector, Ar carrier gas) (Shimadzu, Duisburg, Germany).

## 3. Results

**Figure 1:**
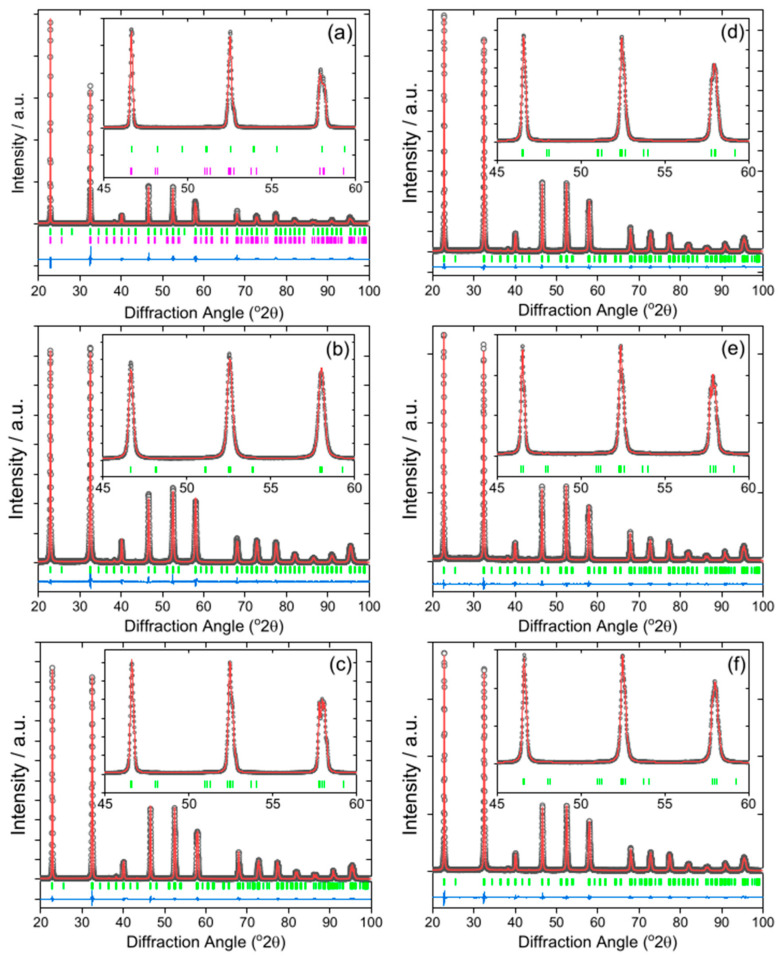
Fitted powder XRD patterns (λ = 1.5418 Å) of Ir-NaTaO_3_ prepared by hydrothermal synthesis with intended Ir content of (**a**) 0% (*R*_w_ = 9.787), (**b**) 10% (*R*_w_ = 6.070), (**c**) 20% (*R*_w_ = 4.969), (**d**) 30% (*R*_w_ = 4.767), (**e**) 40% (*R*_w_ = 6.089), (**f**) 50 % (*R*_w_ = 6.767). The data are the black circles, the red line is the fitted pattern, the blue line isthe difference curve, and the green tick marks are the positions of allowed Bragg reflections (space group, *Pbnm*). In (**a**), the second set of pink tick marks are due to the minor *Cmcm* phase, where a Rietveld fit was used. (**b**–**f**) are Pawley fits.

**Figure 2:**
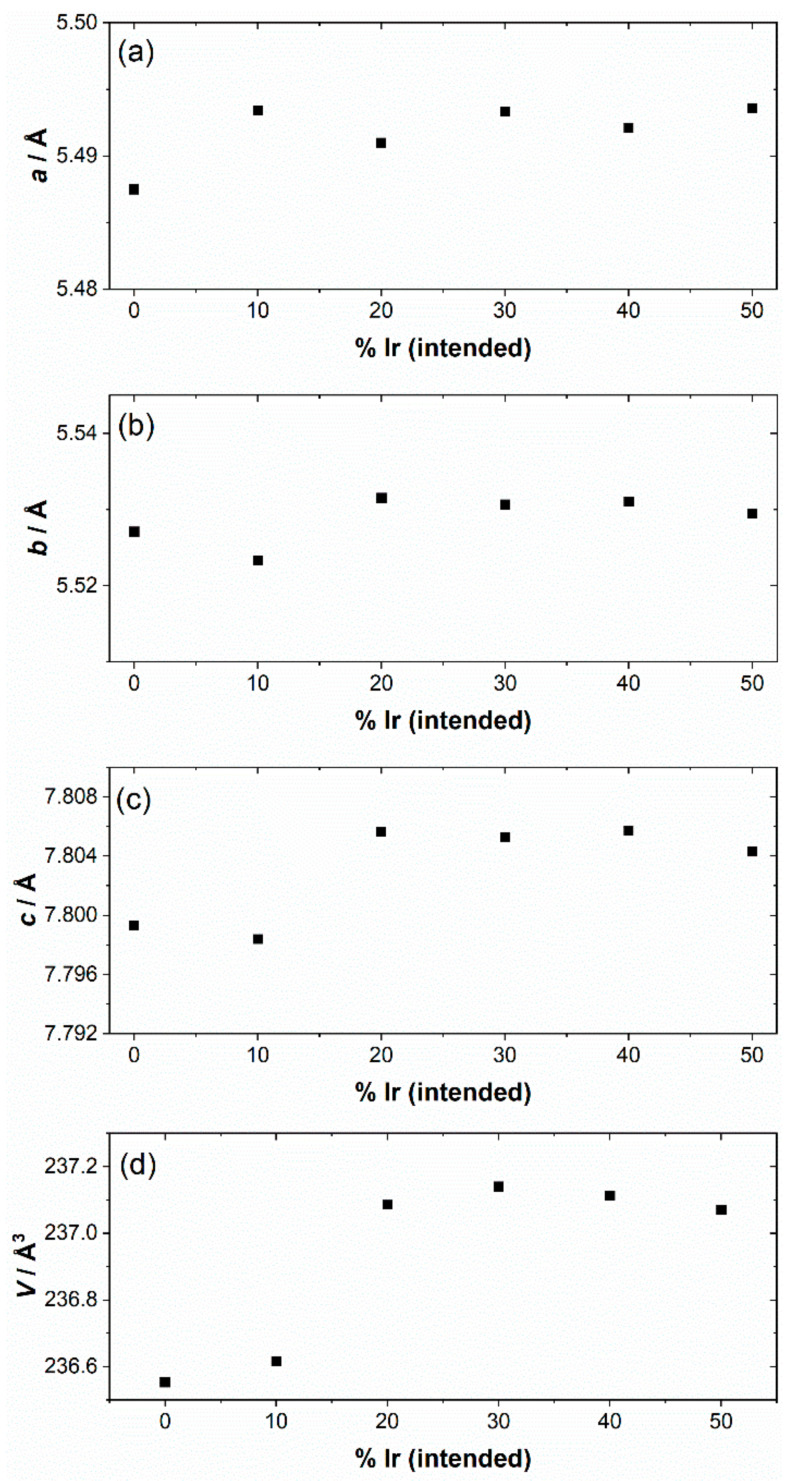
Variation of lattice parameters of Ir-NaTaO_3_ materials with intended composition. (**a**) *a*, (**b**) *b*, (**c**) *c*, and (**d**) unit cell volume. The error bars are smaller than the data points.

The iridium-containing samples are all produced as green powders, in contrast to the white parent NaTaO_3_. The powder XRD pattern of the unsubstituted NaTaO_3_ material was initially fitted using the orthorhombic space group, *Pbnm*, but a closer examination of the pattern revealed some mismatched peak intensities, which could be remedied by inclusion of a second *Cmcm* polymorph. A two-phase Rietveld analysis with atom coordinates and temperature factors fixed at the values reported in the literature [41], gave a satisfactory fit to the data, Figure 1a. The refined lattice parameters are in agreement with the literature values, Table 1, and the mixed-phase nature of NaTaO_3_ has been previously seen, with samples prepared by solid-state synthesis showing ~45% of the *Cmcm* polymorph [38]. The smaller amount of the second polymorph that we observe (24.9%) would be consistent with the different synthesis route that we have used, but it is noteworthy that the hydrothermal method yields a proportion of the *Cmcm* polymorph, that has been defined as a high-temperature phase. The powder XRD patterns of the iridium substituted sodium tantalate materials can all be fitted using the single *Pbnm* polymorph, Figure 1b–f. The variation of lattice parameters with intended Ir content, Table 1 and Figure 2, provides evidence for the inclusion of Ir into the perovskite structure, in particular since all materials with the highest Ir content have larger orthorhombic *c* axes and corresponding larger unit cell volumes. It can be seen that the material with the smallest intended Ir amount has unit cell parameters rather similar to the parent NaTaO_3_. The ionic radius of both Ir^4+^ (0.625 Å) and Ir^5+^ (0.570 Å) in octahedral coordination are similar to Ta^5+^ (0.640 Å) [42]. Given that it is most likely that the tantalum is replaced in the perovskite, we can propose that a smaller Ir content may be associated with the higher oxidation state, while addition of larger quantities results in inclusion of more Ir^4+^, with associated charge-balancing oxide-ion vacancies, and hence modification of the lattice parameters. The iridium oxidation state will be discussed further below in the light of spectroscopic evidence. If larger amounts of iridium were added to the synthesis, then no further inclusion of Ir into the perovskite structure was observed and instead poorly crystalline IrO_2_ was formed as a byproduct.

**Figure 3:**
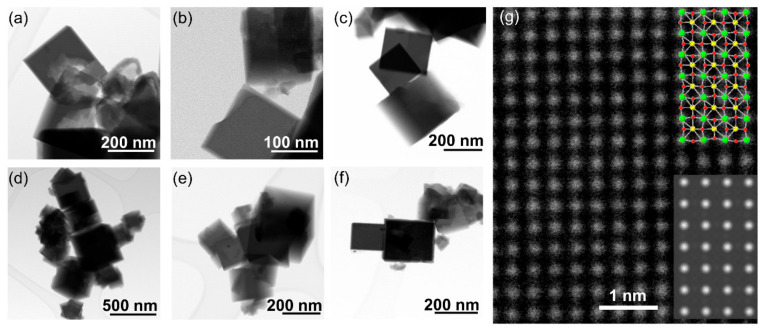
TEM images of Ir-substituted NaTaO_3_ materials with intended Ir content of (**a**) 0%, (**b**) 10%, (**c**) 20%, (**d**) 30%, (**e**) 40% and (**f**) 50%. See Supporting Information for further images of the specimens. (**g**) shows a high-resolution TEM image of the 40% Ir material, with the simulated image (lower inset) and the corresponding crystal structure (upper inset, where green atoms are Ta(Ir), yellow are sodium, and red are oxygen.

**Figure 4:**
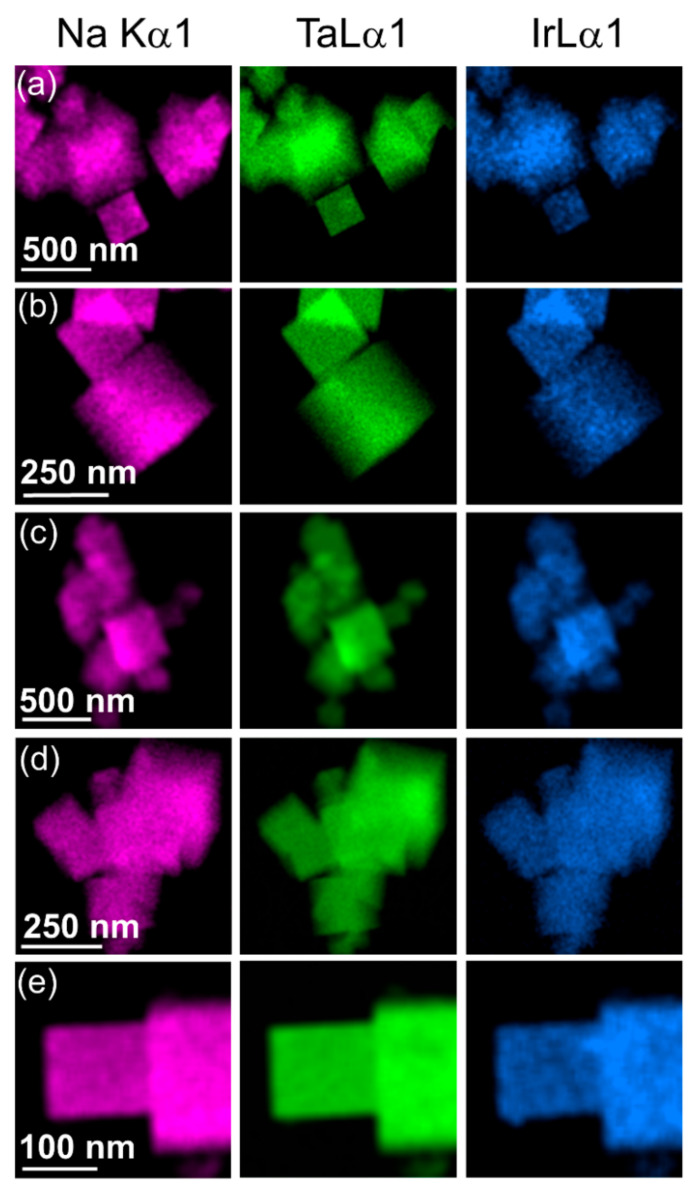
EDS maps performed using TEM of Ir-substituted NaTaO_3_ materials with intended Ir content of (**a**) 10%, (**b**) 20%, (**c**) 30%, (**d**) 40%, and (**e**) 50%.

The iridium-substituted sodium tantalates are formed as nanocubes with edges in the range of up to a few hundreds of nanometres, and typically ~100 nm, as seen by STEM, Figure 3. Although the cubes are not monodisperse in size, they have high crystallinity, as evidenced by high-resolution atomic-scale imaging, Figure 3g. EDS maps measured in STEM show an even dispersion of iridium in the substituted materials, with no evidence of clustering of iridium, nor any distinct particles of separate iridium-rich material, such as iridium oxide, or iridium metal, Figure 4. The cube-shaped particles are similar to hydrothermally synthesised sodium tantalate reported in literature [43,44,45], including those substituted with Bi^3+^ [46] and Cu^2+^ [47].

Quantification of the EDS performed using scanning electron microscopy reveals that the iridium content of the materials is somewhat lower than the amounts used in the reactions used to prepare them, Table 2. This is consistent with the strong colour of the filtrate during washing of the samples, which suggests not all the precious metal was incorporated into the final material. Nevertheless, the amount of iridium does increase proportionally with increasing amount used in synthesis.

**Figure 5:**
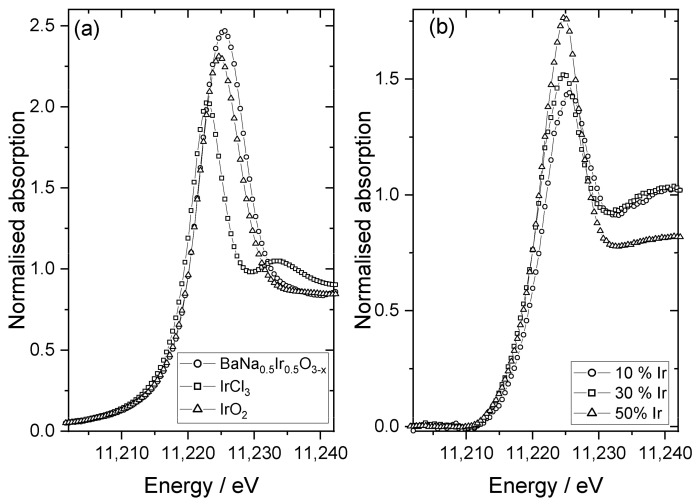
Ir L_III_-edge XANES spectra: (**a**) reference materials for oxidation state calibration and (**b**) Ir-substituted NaTaO_3_ materials labelled according to the proportion of Ir added in synthesis.

**Figure 6:**
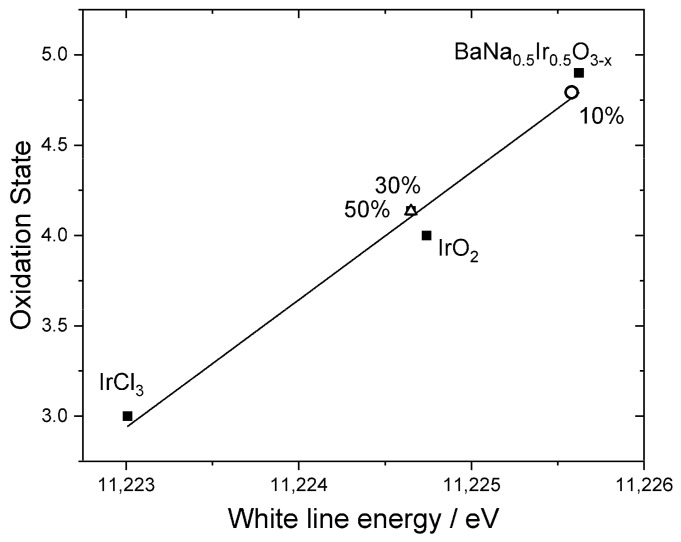
Oxidation states of Ir-substituted NaTaO_3_ materials labelled according to the proportion of Ir added in synthesis, plotted with reference materials. The line is the linear regression fit to the points from the reference materials.

Three representative samples were studied in more detail to understand the chemical state of iridium in the samples. XANES spectra recorded at the Ir L_III_-edge, Figure 5, were used to determine the average oxidation state of iridium by comparison to reference materials. Here, the position of the white line was used as a measure of edge shift, as in our previous work [48], and the materials IrCl_3_, IrO_2_, and BaNa_0_._5_Ir_0_._5_O_3−*x*_ (*x* = 0.525) were used as calibrants for oxidation states +3, +4, and +4.9, respectively. This shows that the iridium in the materials with higher levels of Ir has an oxidation state of close to +4, as in IrO_2_, Figure 6. The sample with the lowest iridium content appears to have a higher average Ir oxidation state, but still lower than +5. The chemistry of Ir^4+^ and Ir^5+^ in oxides is almost exclusively associated with octahedral coordination [49] and so it is anticipated that iridium occupies the B-site of the perovskite structure.

The results of the XANES analysis are consistent with the results from powder XRD, as described above. The sample with the smallest amount of iridium contains a significant proportion of Ir^5+^, which would be consistent with the small changes in lattice parameter, but the materials with greater Ir content contain Ir^4+^, which is larger in ionic radius and would require charge-balancing oxide-ion vacancies, and hence rather different lattice parameters. The reason for this difference is not apparent from the data we have measured, and it may be the case that a greater surface accumulation of Ir at the higher amounts used leads to a change in average oxidation state of iridium, or that the redox chemistry in solution leads to only small amounts of Ir^5+^ that are available for inclusion in the perovskite structure.

**Figure 7:**
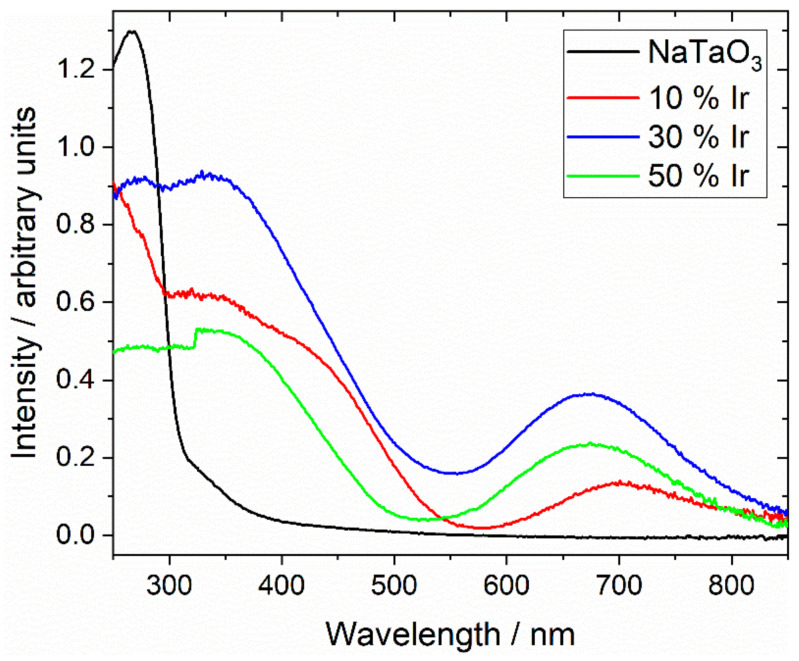
Diffuse-reflectance UV-vis spectra of Ir-substituted NaTaO_3_ materials labelled according to the proportion of Ir added in synthesis.

Figure 7 shows diffuse-reflectance UV-vis spectra measured from the same three samples, along with the spectrum of NaTaO_3_ for comparison. The parent perovskite NaTaO_3_ shows a spectrum very similar to that reported from the literature [50], and a Tauc plot analysis gives a direct band gap of 4.0 eV, as expected. The iridium-containing materials show significant absorption in the visible part of the spectrum, which illustrates the green colour of the powdered samples, with strong absorptions in the 450 nm and 700 nm regions. Interestingly, the sample with smallest iridium content shows different absorption maxima to the samples with the higher iridium content, suggesting a different electronic state for the iridium cations in the solid. This is entirely consistent with the XANES and powder XRD analysis presented above.

**Figure 8:**
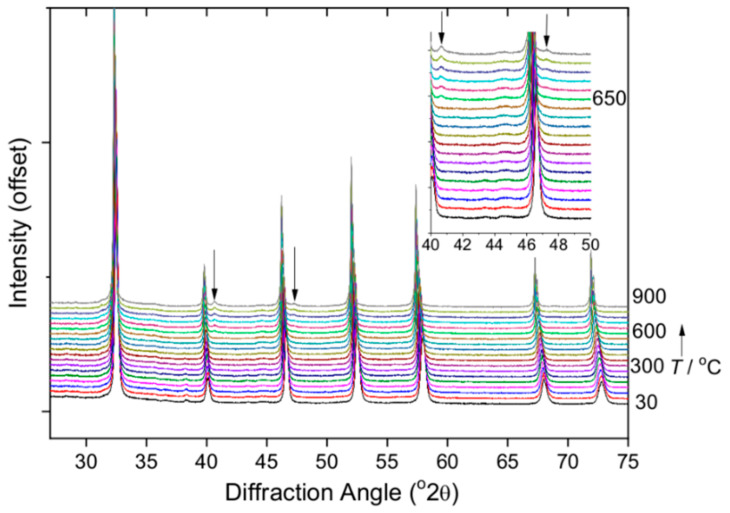
Variable temperature XRD on heating in air showing the stability of Na(Ta,Ir)O_3_ until above 600 °C when iridium metal is seen, with its two strongest Bragg peaks, (111) and (200), indicated by the arrows.

We examined the materials use in photocatalysis by studying hydrogen generation from water using the material with highest iridium content. To optimise the activity, the powders were heated in air at 500 °C to remove any surface-bound water and minimise any possible hydroxyl defects that may be anticipated in hydrothermally produced oxide perovskites [51,52,53]. To verify that this did not result in phase separation, X-ray powder thermodiffractometry was carried out with heating from room temperature to 900 °C, Figure 8. This shows that the perovskite structure remains unchanged until above 600 °C and only then are the strongest Bragg peaks of face-centred cubic iridium metal [54] observed.

**Figure 9:**
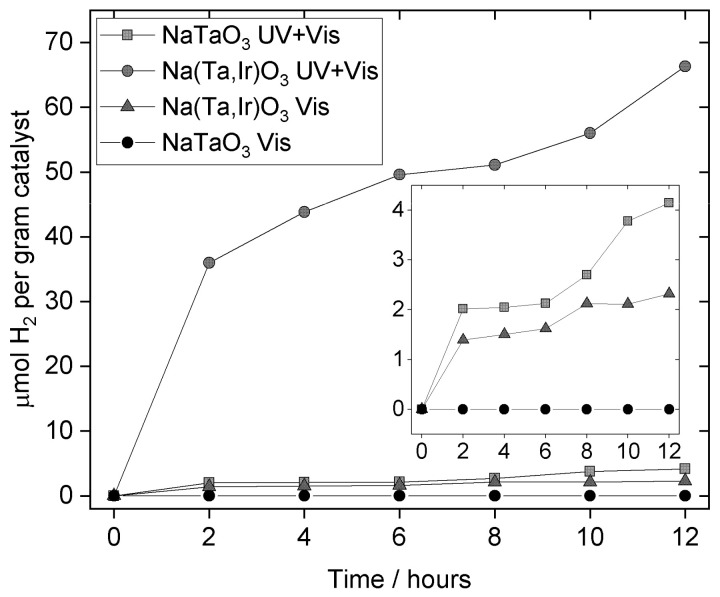
Photocatalytic hydrogen evolution observed from NaTaO_3_ and Ir-substituted sample.

Photocatalysis results reveal that the parent NaTaO_3_ when loaded with 1 wt.% Pt shows a low activity in full-spectrum irradiation, but no detectable activity under visible light towards hydrogen evolution in aqueous methanol, Figure 9. In contrast, the full-spectrum irradiation of Na(Ta,Ir)O_3_ yields approximately 15 times the yield of hydrogen, and, notably, under visible light shows yields of hydrogen comparable to the un-substituted material in UV + visible light. These preliminary results demonstrate the effectiveness of iridium substitution in tuning optical properties of NaTaO_3_ to provide visible light activity. We note that the catalytic production of hydrogen is not linear, but this has been reported previously for Bi-containing NaTaO_3_ studied under the same conditions [55]. The cause of this is not known at present, but may be related to a change of the catalyst surface with time, such as restructuring of surface defects.

## 4. Discussion

The observation of iridium in oxidation states between +4 and +5 in the perovskite structure is consistent with other iridium oxides that contain octahedrally coordinated Ir and that have been prepared under similar hydrothermal conditions in alkali solutions in the presence of peroxide as oxidant [35,48]. It is noteworthy that for the material with the lowest iridium concentration a higher oxidation state is observed spectroscopically, while increasing Ir concentration lowers the average Ir oxidation state (corroborated indirectly by powder XRD). Interestingly, for other iridium-substituted perovskites reported in the literature via other synthesis methods, different oxidation states of Ir may be found. For example, Calì et al. observed Ir^3+^ using X-ray photoelectron spectroscopy in 5 mol% Ir-substituted SrTiO_3_ that had been prepared by solid-state synthesis at 1340 °C [31]. On the other hand, Kawasaki et al. found Ir^4+^ in samples of Ir-substituted SrTiO_3_ prepared by solid-state synthesis at 1100 °C [32]. While the valence states of the host perovskite material might influence the Ir oxidation state, the synthesis method is likely to play an important role, and hence the hydrothermal method offers an alternative approach to solid-state synthesis that might allow a more tunable synthesis of Ir-containing perovskites.

The presence of Ir^4+^ in NaTaO_3_ means that charge-balancing defects must be necessary. In other precious-metal-doped perovskites, surface oxide defects have been inferred, for example, in Ir-SrTiO_3_ [32]. In the case of NaTaO_3_ prepared by hydrothermal synthesis, inclusion of Bi^3+^ was accompanied by oxide defects, based on evidence from photoluminescence spectra [46]. It is also a possibility that the hydrothermal route allows charge-balance by inclusion of hydroxide ions in place of oxide: these have been detected in other perovskites prepared by this method, such as Na_0_._5_Bi_0_._5_TiO_3_ [51,53] and KNbO_3_ [52]. Defects such as anion vacancies may be in part responsible for the enhanced light absorption and photocatalytic activity of the substituted materials [56]. Further in-depth experimental work is needed to explore the role of defects and how these are modified in the presence of the co-catalyst, and with heat treatment. Methods such X-ray photoelectron spectroscopy would be useful to example the nature of oxide defects, such as lattice vacancies [57].

The materials we have prepared show stability on heating in air to only 600 °C, which demonstrates how it would be impossible to prepare the same samples by conventional solid-state synthesis, or even coprecipitation methods that require an annealing step. It is interesting to note that Rh^3+^-containing BaTiO_3_ prepared by an oxalate-aided co-precipitation route showed extrusion of rhodium metal when heated above its synthesis temperature [30]. This illustrates the tendency for the precious metals to be reduced, but also how this may be aided by embedding in a host lattice by replacement of a cation of higher charge.

Our photocatalysis results show the beneficial effect of Ir-inclusion in NaTaO_3_ with greater than ten-fold enhancement of activity towards hydrogen evolution in UV+visible radiation, a significant part of which can be ascribed to visible light absorption. The results obtained are a similar order of magnitude to those produced from Bi^3+^-NaTaO_3_, studied using the same experimental protocol [55], although they do not reach such a high hydrogen yield. However, there is scope for further optimisation, as the previous work showed how Pt loading and substitution level of the perovskite should be adjusted to improve yield, and that the surface area of the materials should be controlled for maximum catalytic efficiency. In optimising photocatalytic properties for hydrogen evolution of Ir–SrTiO_3_ the oxidation state of Ir is important [32], but also loading with surface Ir metal can optimise properties [58]. Hence, our work provides a convenient synthesis method that may allow further tuning of properties of materials for visible-light photocatalysis.

## 5. Conclusions

We have presented a hydrothermal synthesis route to introduce iridium into a prototypical perovskite structure NaTaO_3_ that uses mild reaction conditions in a single-step process. A comprehensive set of characterisation data shows that the iridium replaces tantalum to give small adjustments in lattice parameters. For the samples that contain the most iridium, the substituent is present in the +4 oxidation state, which implies the presence of defects for charge balance, but this is in line with other perovskites that contain precious-metal substituents, and for other oxides that have been prepared by hydrothermal chemistry. Our preliminary photocatalysis results show promising properties for visible-light generation of hydrogen from water, but beyond this the materials may prove useful for other applications in heterogeneous catalysis, either as prepared, or upon reduction to extrude the iridium as supported nanocrystals. Finally, we note that the redox chemistry taking place during synthesis as the Ir^3+^ precursor is oxidised is likely to be complex, and this is where the origin of the substitutional chemistry occurs. Further work is needed to understand the mechanisms of hydrothermal crystallisation of oxides, and the evolution of the solution chemistry as a solid is formed is largely unexplored at present.

## Figures and Tables

**Table 1:** Refined lattice parameters of substituted sodium tantalates from analysis of powder XRD data. The intended level of Ir substitution is indicated.

Material	Lattice Parameters		
	*a*/Å	*b*/Å	*c*/Å
NaTaO_3_ [41]	5.48109(9)	5.52351(9)	7.79483(12)
NaTaO_3_^a^	5.48750(5)	5.52711(6)	7.7993(1)
NaTaO_3_-10% Ir	5.4934(1)	5.5233(1)	7.7984(2)
NaTaO_3_-20% Ir	5.49099(4)	5.53154(5)	7.8056(6)
NaTaO_3_-30% Ir	5.49335(6)	5.53067(9)	7.8053(1)
NaTaO_3_-40% Ir	5.49212(8)	5.53101(9)	7.8057(1)
NaTaO_3_-50% Ir	5.4936(1)	5.5295(1)	7.8043(1)

^a^: The major (75.1%) *Pbnm* phase is shown, the second *Cmcm* phase has lattice parameters *a* = 7.795(2) Å, *b* = 7.789(3) Å, *c* = 7.791(5) Å (cf. literature values [38], *a* = 7.77927(8) Å*, b* = 7.7815(2) Å, *c* = 7.7899(1) Å).

**Table 2:** EDXA of Ir-substituted NaTaO_3_ materials performed using scanning electron microscopy.

	EDS Results		
Intended Ir Substitution	Tantalum/%	Iridium/%	Determined Formula
10%	97.6	2.4	NaTa_0_._98_Ir_0_._02_O_3_
20%	95.3	4.7	NaTa_0_._95_Ir_0_._05_O_3_
30%	91.6	8.4	NaTa_0_._92_Ir_0_._08_O_3_
40%	87.8	12.2	NaTa_0_._88_Ir_0_._12_O_3_
50%	85.1	14.9	NaTa_0_._85_Ir_0_._15_O_3_

## Data Availability

The research data underpinning this article can be accessed at: http://wrap.warwick.ac.uk/153846/ (accessed on 9 June 2021).
